# Would artificial neural networks implemented in clinical wards help nephrologists in predicting epoetin responsiveness?

**DOI:** 10.1186/1471-2369-7-13

**Published:** 2006-09-18

**Authors:** Luca Gabutti, Nathalie Lötscher, Josephine Bianda, Claudio Marone, Giorgio Mombelli, Michel Burnier

**Affiliations:** 1 Division of Nephrology, Ospedale la Carità, Via Ospedale, 6600 Locarno, Switzerland; 2 Roche Pharma Switzerland Ltd, Reinach, Switzerland; 3 Department of Internal Medicine, Ospedale San Giovanni, Bellinzona, Switzerland; 4 Department of Internal Medicine, Ospedale la Carità, Locarno, Switzerland; 5 Division of Nephrology, University Hospital of Lausanne, Lausanne, Switzerland

## Abstract

**Background:**

Due to its strong intra- and inter-individual variability, predicting the ideal erythropoietin dose is a difficult task. The aim of this study was to re-evaluate the impact of the main parameters known to influence the responsiveness to epoetin beta and to test the performance of artificial neural networks (ANNs) in predicting the dose required to reach the haemoglobin target and the monthly dose adjustments.

**Methods:**

We did a secondary analysis of the survey on Anaemia Management in dialysis patients in Switzerland; a prospective, non-randomized observational study, enrolling 340 patients of 26 centres and in order to have additional information about erythropoietin responsiveness, we included a further 92 patients from the Renal Services of the Ente Ospedaliero Cantonale, Bellinzona, Switzerland. The performance of ANNs in predicting the epoetin dose was compared with that of linear regressions and of nephrologists in charge of the patients.

**Results:**

For a specificity of 50%, the sensitivity of ANNs compared with linear regressions in predicting the erythropoietin dose to reach the haemoglobin target was 78 vs. 44% (*P *< 0.001). The ANN built to predict the monthly adaptations in erythropoietin dose, compared with the nephrologists' opinion, allowed to detect 48 vs. 25% (*P *< 0.05) of the patients treated with an insufficient dose with a specificity of 92 vs. 83% (*P *< 0.05).

**Conclusion:**

In predicting the erythropoietin dose required for an individual patient and the monthly dose adjustments ANNs are superior to nephrologists' opinion. Thus, ANN may be a useful and promising tool that could be implemented in clinical wards to help nephrologists in prescribing erythropoietin.

## Background

Stable haemoglobin levels maintained in the target range of 11 to 12 g/dL as recommended by the Kidney Disease Outcomes Quality Initiative, are associated with both clinical and quality of life benefits as well as a reduction in hospitalisation and mortality [1-4]. Whether, in targeted subgroups, the haemoglobin concentration should be set above 12 g/dL has not been definitively demonstrated [5]; however, considering the likelihood of increasing thrombotic events [6,7], its value should not exceed 14 g/dL [5].

The response to erythropoietin is known to have a large inter- and intra-individual variability explained by blood losses, co-morbidities [8], dialysis efficiency [9,10], iron status [5,11], folic acid and vitamin B12 deficiency [12], hyper- and hypo-parathyroidism [13,14], pro-inflammatory cytokine activities [15], aluminium toxicity [14], treatment with angiotensin converting enzyme inhibitors (ACE-I) [16] and probably angiotensin II receptor blockers (ARB) [17]. Thus, maintaining the haemoglobin level in the target range is sometimes a difficult task which necessitates regular doses adjustments.

To optimize anaemia management several protocols, based on physician or nurse-driven algorithm as well as computer assisted prescription tools, some of them involving the use of Artificial Neural Networks (ANNs), have been described [18-26].

A large amount of clinical and biochemical data that could be useful in making crucial follow-up decisions are actually collected during dialysis sessions [27-29]. Unfortunately the multidimensionality and at least partial non-linearity of the data, (i) limits the value of both intuition/experience of the nephrologists and standard statistical procedures and (ii) makes their interpretation and practical use in clinical wards difficult [27]. The importance of individualizing drug dosage regimens by adding patient-specific post-administration data about serum levels or responsiveness to population pharmacokinetic and dynamic models, has been thoroughly demonstrated [30]. Compared to other non linear mathematical and statistical tools based for instance on Bayesian fitting and adaptive control, ANNs have the advantage of being user friendly, tolerating missing data and errors in individual variables well and also of being applicable to translate multivariate non-linear relationships into continuous functions without the need of understanding precisely the underlying relationships between variables [31-36]. ANNs have been widely used in clinical medicine and have already assisted nephrologists in solving various complex clinical problems [27-33,37,38].

The purposes of the present study were (i) to characterize the linear or non-linear relationships between several clinical and biological variables and the response to epoetin beta and (ii) to build a computer assisted mathematical tool able to predict the epoetin requirement in an individual patient and the monthly adjustments in the epoetin dose.

## Methods

### Patient characteristics

We did a secondary analysis of the survey on Anaemia Management in dialysis patients in Switzerland (AIMS); a prospective, open label, non-randomized observational study designed to assess anaemia management in the dialysis centres in Switzerland [Lötscher N, et al. Swiss Med Forum 2004; 4: S7; Abstract]. In this study (inclusion and exclusion criteria were: current dialysis treatment, age > 18 y, renal anaemia requiring epoetin therapy, ferritin > 200 μg/L and respectively absence of vitamin B12 or folic acid deficiency, unstable angina pectoris, untreated hypertension, haemoglobinopathy, haemolysis, epilepsy), data about sex, weight, age, presence or absence of a diabetes mellitus and/or a cardiomyopathy, haemoglobin, epoetin beta dose and its administration route (subcutaneous vs. intravenous), ferritin and creatinine were collected in 340 hemodialysis patients. In order to increase the statistical power of the study and to gain more information about factors influencing erythropoietin responsiveness, a further 92 haemodialysis patients (with complete historical data and selected with the same criteria applied for the AIMS survey) were included in the analysis. These additional patients were selected based on the same inclusion criteria from the Renal Services of the Ente Ospedaliero Cantonale (*EOC*), Bellinzona, Switzerland after having been followed for at least 12 months (the 2 databases pooled together being called AIMS*EOC *data). For each patient the sex, weight and age, the haemoglobin, epoetin beta dose and its administration route (subcutaneous vs. intravenous), ferritin, creatinine, urea, Kt/V, pH, phosphate, ionized calcium, albumin, parathyroid hormone (PTH), C reactive protein (CRP) and intravenous iron dose over the 12 treatment months were collected. Comorbidity information about the concomitance of diabetes mellitus, cardiomyopathy with impaired left ventricular ejection fraction (<50%) and ACE-I or ARB medication were also registered.

### Kt/V calculation

The Kt/V, a parameter of dialysis adequacy defined as the dialyzer clearance of urea multiplied by the duration of the dialysis treatment and divided by the volume of distribution of urea in the body, was estimated with a second generation single-pool Daugirdas formula [39]: Kt/V = -ln (R -0.03) + [(4-3.5 × R) × (UF/W)] where: R = post-dialysis urea/pre-dialysis urea, UF = net ultrafiltration and W = weight.

### Estimation of the erythropoietin dose required to obtain a haemoglobin of 11.5 g/dL

To calculate the epoetin dose that should have been prescribed in order to obtain a haemoglobin of 11.5 g/dL a linear regression plotting epoetin dose against haemoglobin was built for each patient. The choice of using suboptimal tools like linear regressions to estimate the ideal epoetin dose was made considering that (i) the small number of observations reduced the statistical options at our disposal and (ii) an epoetin dose approximating the ideal one was necessary to build the models.

### Artificial Neural Networks

In order to build the non-linear continuous functions expressing the interdependency between the collected data and the epoetin dose a series of artificial neural networks (ANNs) were built, trained, cross-validated and tested using the NeuroSolution for Excel 4.32 software, NeuroDimension Inc.

**Figure 1:**
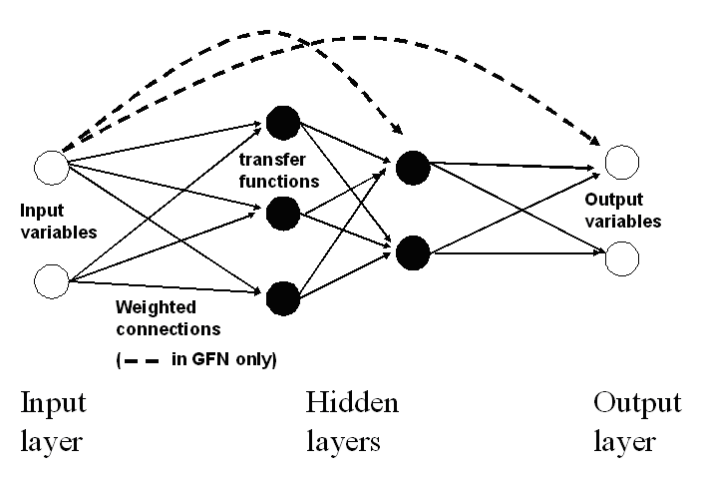
**Schematic representation of an artificial neural network**. A typical ANN consisting of one input layer, two hidden layers and one output layer is represented. The basic structure, fed forwards and trained by back-propagation is called Multilayer Perceptron (MLP) while models designed with connections jumping over hidden layers (---) are called Generalized Feedforward Networks (GFN).

ANNs are composed of one input layer (collecting input variables expected to be predictive), one output layer (collecting the predictions, known in training and unknown in testing and validation cases) and one or more hidden layers (performing a weighted sum of the inputs and passing the resulting value through a non-linear function to the output layer). Individual weights are progressively adapted, using for instance a back-propagation algorithm, to minimize the difference between calculated and expected outputs; the weights assuring the best results then being used to test and compare the performance of the ANNs (see Figure 1 for a schematic representation).

The databases selected to be analyzed were randomized using the same software package. After selecting 25% of the data for the validation phase, the remaining pool was divided into the training, cross validation and testing subgroups assigning respectively to each one 50, 10 and 40% of the data. As a network structure only Multilayer Perceptrons (MLP) (layered feed-forward networks trained with static backpropagation) and Generalized Feedforward Networks (GFN) (generalization of the MLP with connections jumping over hidden layers) were used (see Figure 1). Seeing that the critical difference between the two network structures is the amount of training data requested to optimize the performance (higher in the MLP and lower in the GFN) and because of the database with a limited extension used, each set of variables was used to train both models. A hyperbolic tangent transfer function (maximum and minimum output 1 and -1 respectively), as recommended by the producer (default setting), was chosen for each neuron. All initial connection weights were randomized before beginning a training phase. As a learning rule a gradient and weight change one (momentum) was chosen. To avoid overtraining, the training phase was stopped when the minimum squared error (MSE), displayed as a function of the training epochs, between the predictions and the desired output in the cross validation subgroup (indirect indicator of the level of generalisation) began to increase. All the networks were built using only one hidden layer while the ideal number of hidden neurons was determined by training, cross validating and testing a MLP, increasing unitarily the number of neurons beginning with 4 and stopping when the MSE between the predictions and the desired output in the testing subgroup began to increase. Once a definitive MLP was obtained, its performance was compared with a GFN with an identical number of processing elements (being both trained, cross validated and tested 5 times) and the best performing model (best compromise in the testing subgroup between linear correlation and normalized mean squared error *r*/NMSE) (see "Statistical and data analysis" paragraph for details) was selected to be tested in the validation subgroup. All studied variables were first analyzed separately and then combined in order to achieve the best prediction performance. The study was designed according to the prescriptions of Cross et al. [33].

### Prediction by the nephrologists in charge of the patients of a follow-up haemoglobin level below the target of 11.0 g/dL

The sensitivity, specificity and the positive and negative predictive values in detecting a follow-up haemoglobin < 11.0 g/dL were calculated by interpreting the decision of the nephrologists to increase the epoetin dose as if it had been a prediction of a follow-up haemoglobin below the target level. Respecting statistical and modeling exigencies the haemoglobin measured one month after the adaptation in the dose has been considered as the follow-up haemoglobin.

### Selection of the data for the prediction of the monthly epoetin dose adjustments

The data necessary to build and test the ANN predicting the monthly epoetin dose adjustments were selected from the AIMS*EOC *pool. Complete data of 4 consecutive months from months 3 to 6 and/or 7 to 10 were requested. The data of the months 3–5 and 7–9 were used as input while the data of months 6 and 10 were used as desired output.

### Statistical and data analysis

Statistical and data analysis was performed using a statistical software package (SPSS 12.0; SPSS Inc.). SPSS was also used to build linear regressions (forward stepwise method based on the F probability including collinearity diagnostics) and to infer receiver-operating-characteristic curves (ROC) plotting the sensitivity against 1 minus the specificity for each prediction from the ANN, the linear regressions or the nephrologists (the areas under the curves were calculated by the trapezoidal nonparametric method and are expressed with the 95% confidence interval). Accuracy was expressed by the Combined Root Mean Square Error (CRMSE) calculated as the square root of [(mean difference in estimate-observed)^2 ^+ (standard deviation of the difference)^2^]. Agreement between the predictions and the basis data was expressed by "limits of agreement", "95% confidence interval for the bias" and "95% confidence interval for the lower and upper limits of agreement" according to Bland and Altman [40]. The mean difference in estimate – observed, also called "bias", and the standard deviation of the difference of the same subtraction, also called "precision" are included concepts in both CRMSE and "limits of agreement". Histograms comparing the performance of the ANNs and the best performing linear regression were built using Excel SP3, Microsoft Inc. With the intention of facilitating the graphical representation of the differences in the predictive performances the ratio *r*/NMSE was calculated dividing the Pearson linear correlation coefficient *r *by the normalized mean squared error (obtained dividing the mean squared error by the variance of the reference population); the higher the value the better the performance. Percentages were compared using a Fisher exact test. The values are in mean ± standard deviation (SD). A *P*-value < 0.05 was considered statistically significant.

## Results

### Population characteristics

The 12-month follow-up of the AIMS survey was initiated in June 2002. 340 patients from 26 centres were included in the final analysis. The mean age of the participating patients was 63 ± 15 y. The most common causes of end-stage renal disease were glomerulonephritis (23%), diabetic nephropathy (21%) and hypertension (21%). The most prevalent baseline co-morbidities were cardiac-related. Hypertension occurred in 61% of the patients and diabetes was reported in 27%. The mean haemoglobin concentration was 11.8 ± 1.4 g/dl with approximately 80% of the included patients achieving the target haemoglobin of at least 11 g/dL. The mean epoetin beta dose was 149 ± 104 IU/kg/week.

Ninety-two patients (1104 monthly clinical and biochemical data) of the 3 dialysis units of the renal services of the Ente Ospedaliero Cantonale (*EOC*), Bellinzona, Switzerland, all meeting the requisite criteria, were also included in the analysis.

The basic information including comorbidity incidence, ACE-I or ARB treatment and mean results of the monthly biochemical parameters of the AIMS and *EOC *data pools are listed in Table 1 (recurrent abbreviations in the tables and figures are summarized in Table 2).

**Table 1:** Characteristics of the studied populations

	**AIMS data (± SD)**	***EOC *data (± SD)**
	
**N**	340	92
**Sex (% of males)**	58.2	45.6
**Age (y)**	63.5 ± 14.6	69.7 ± 12.1
**Weight (kg)**	69.5 ± 15.3	69.9 ± 15.4
**Diabetes mellitus (%)**	27.4	34.7
**Cardiomyopathy (%)**	16.5	13.0^a^
**ACE-I or ARB treatment (%)**	*No data*	89.1
**Haemoglobin g/dL**^**b**^	11.8 ± 1.4	11.6 ± 0.7
**Creatinine μmol/L**^**b**^	580 ± 156	552 ± 145
**BUN mmol/L**^**b**^	*No data*	24.6 ± 6.0
**Kt/V**	*No data*	1.33 ± 0.26
**pH**^**b**^	*No data*	7.36 ± 0.04
**Phosphate mmol/L**^**b**^	*No data*	2.00 ± 1.38
**Ionized calcium mmol/L**^**b**^	*No data*	1.20 ± 0.07
**Albumin g/L**^**b**^	*No data*	37.9 ± 2.9
**Ferritin mg/mL**	411 ± 297	482 ± 255
**PTH pmol/L**	*No data*	25.8 ± 21.9
**CRP mg/L**^**b**^	*No data*	15.7 ± 15.8
**Iron intravenously mg/month**	*No data*	134 ± 50
**Epoetin beta dose IU/Kg/week to reach a haemoglobin of 11.5 g/dL**	149 ± 104	107 ± 63
**Epoetin administration route % of subcutaneous**	71	0

^a ^only patients with a left ventricular ejection fraction <50%; ^b ^pre-dialysis values

Anthropometric data, comorbidity incidence, ACE-I or ARB treatment and mean results of the monthly biochemical parameters of the AIMS and *EOC *data pools.

**Table 2:** Abbreviations

Anaemia Management in dialysis patients in Switzerland	**AIMS**
Renal units of the Ente Ospedaliero Cantonale, Bellinzona, Switzerland	***EOC***
AIMS and EOC data pooled	**AIMS*EOC***
Artificial neural network	**ANN**
Linear regression	**LIN REG**
Nephrologist	**NEPH**
Sex	**SEX**
Age	**AGE**
Weight	**W**
Diabetes mellitus	**DM**
Cardiomyopathy	**CARDIO**
Angiotensin converting enzyme inhibitor or angiotensin receptor blocker treatment	**ACE/ARB**
Haemoglobin	**HB**
Creatinine ^a^	**CREA**
Blood urea nitrogen ^a^	**BUN**
Kt/V	**KTV**
pH ^a^	**PH**
Phosphate ^a^	**PO4**
Ionized calcium ^a^	**CA**
Albumin ^a^	**ALB**
Ferritin	**FERR**
Parathyroid hormone	**PTH**
C reactive protein	**CRP**
Iron intravenously	**IRON**
Epoetin administration route subcutaneous vs. intravenous	**SC/IV**
normalized mean squared error divided by the Pearson correlation coefficient	**NMSE/r**

^a ^pre-dialysis values

Summary of the abbreviations recurrent in the tables and figures

### Intra- and inter-individual variability of the epoetin dose

In the 2 databases pooled together (AIMS*EOC *data) the intra- and inter-individual variability in the epoetin dose prescribed, expressed by the mean absolute deviation from the mean, were 24.7 ± 27.1 and 49.8 ± 48.0 U/Kg/week (*P *< 0.001) respectively.

### Linear correlations between variables and epoetin dose

In the same data pool the variables correlating significantly in a multiple linear regression with the epoetin dose (after intra- or extrapolation for a haemoglobin of 11.5 g/dL) were weight (standardized coefficient beta (β): -1.673; p < 0.001), ferritin (β: 0.079; *P *< 0.001), age (β: -0.800; *P *< 0.05), epoetin administration route (subcutaneous vs. intravenous) (β: -22.730; *P *< 0.05) and presence or absence of a cardiomyopathy (β: 33.050; *P *< 0.01). The obtained linear model with a constant of 301.686 (*P *< 0.001) explained 40.3 % of the variability in the epoetin dose (1.6 % imputable to the epoetin administration route; the intravenous one being associated with an epoetin dose 22.73 U/Kg/week higher). Taking the hemoglobin (before the intra- or extrapolation process) as a dependent variable, the epoetin dose (β: -0.003) and the epoetin administration route (subcutaneous vs. intravenous) (β:-0.528) were the only two significant variables (*P *< 0.001 in both cases) in building a linear model (constant: 12.980, R: 0.405). The epoetin administration route explained 10.2% of the haemoglobin variability in the model; the intravenous one being associated with a lower haemoglobin by 0.528 g/dL.

### Non-linear correlations between variables and epoetin dose

**Figure 2:**
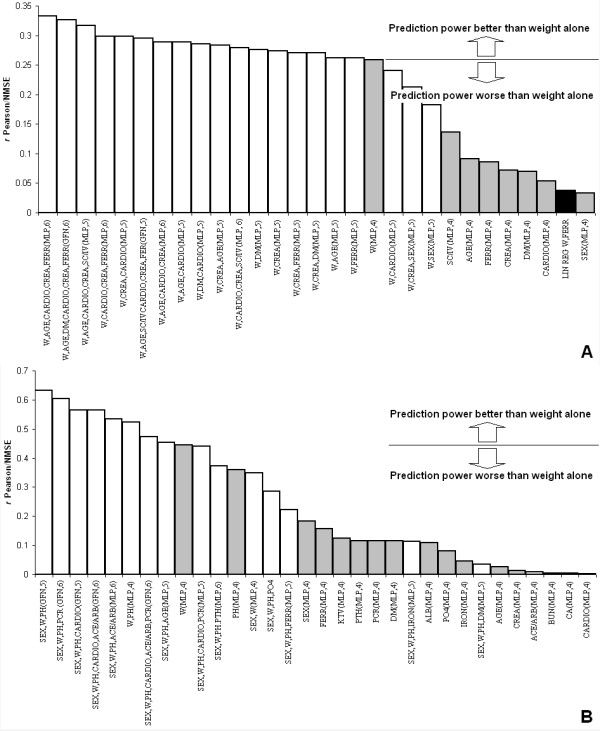
**Performance ability of individual and combined variables in predicting the epoetin dose**. Performance ability of individual and combined variables in predicting the mean epoetin beta dose required for an individual patient to reach the haemoglobin target of 11.5 g/dL using ANNs and linear regressions. The performance ability is expressed by the *r*/NMSE (the higher the value the better the performance). The network structure (either Multilayer Perceptron (MLP) or Generalized Feedforward Network (GFN) and number of processing elements in the hidden layer) is specified in the label of the ANN used for the prediction. Panel A: data from the AIMS*EOC *(training, cross-validation, testing and validation data pool: 170, 30, 122 and 110 patients respectively); the column of the linear regression is in black; individual variables are highlighted in grey. Panel B: data from the *EOC *alone (training, testing and validation data pool: 60, 10 and 22 patients respectively).

The results of the data analysis, performed with both the AIMS*EOC *and the *EOC *data to evaluate the non-linear impact of individual variables in the prediction of the mean epoetin dose required for an individual patient to reach the haemoglobin target of 11.5 g/dL are depicted graphically in Figure 2 (Panels A and B). In both Panels the prediction power of individual and grouped variables is compared in a performance gradient using the ratio between the correlation *r *and the normalized mean square error (the higher the value the better the performance). In Panel A the prediction power of the variables of the AIMS*EOC *data pool is compared with the result of the best performing linear regression (based on weight and serum ferritin). In Panel B the prediction power of the variables of the EOC data pool is shown. The final network structure (either Multilayer Perceptrons (MLP) or Generalized Feedforward Networks (GFN) and number of processing elements in the hidden layer) is specified, in both figures, in the label of the ANN used for the prediction.

### Prediction of the epoetin requirement in an individual patient

The linear regressions, performed for each individual patient, plotting epoetin dose against haemoglobin with the aim of estimating the epoetin dose that should have been prescribed in order to obtain a haemoglobin of 11.5 g/dL, allowed, with a *r *value of 0.247 ± 0.237, to obtain an intrapolated value in 91% of the patients and an extrapolated one in 9%.

**Figure 3:**
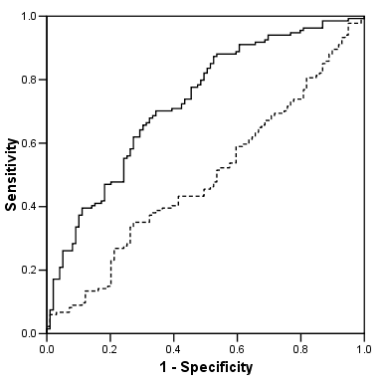
**Prediction of the epoetin dose required to reach the haemoglobin target**. ROC curves plotting sensitivity against 1 minus specificity for a epoetin dose cut-off of 100 IU/Kg/week in the prediction of the dose required for an individual patient to reach the haemoglobin target of 11.5 g/dL obtained from the best performing *linear regression *(*dotted line*; using as input variables weight and ferritin) and the best performing *ANN *(*continuous line*; using as input variables weight, age, presence or absence of an impaired left ventricular ejection fraction, serum creatinine and ferritin). The areas under the curves, the 95% confidence intervals and the significance *P *for the linear regression and the ANN were respectively: 0.491 (0.416–0.565), *P*:n.s. and 0.728 (0.663–0.794), *P *< 0.001 (*P *< 0.001 for the difference between the two curves).

The prediction ability of the best performing linear regression (using as input variables weight and ferritin; β:-1.865 and 0.113 respectively; *P *< 0.001 for both; R:0.431; constant: 235.141) and ANN (using as input variables weight, age, presence or absence of an impaired left ventricular ejection fraction, serum creatinine and ferritin) is further compared using ROC curves in Figure 3 (epoetin dose cut-off 100 IU/Kg/week).

### Prediction of the monthly epoetin dose adjustments

For the prediction of the monthly epoetin dose adjustments required to maintain the haemoglobin in the target range the training, cross-validation, testing and validation data pool consisted respectively of 200, 40, 140 and 110 monthly clinical and biochemical data taken from the AIMS*EOC *pool, including for each individual the haemoglobin and epoetin dose of the two previous months and the haemoglobin of the following one. The mean haemoglobin from the current observation (Hb), the 2 previous (Hb-1 and Hb-2) and the following (Hb+1) month and the mean epoetin dose from the current observation (EPO) and the 2 previous (EPO-1 and EPO-2) months were as follows: Hb: 11.51 ± 1.14, Hb-1: 11.55 ± 1.14, Hb-2: 11.54 ± 1.13, Hb+1: 11.52 ± 1.04, EPO: 83.97 ± 60.67, EPO-1: 87.05 ± 60.15, EPO-2: 85.34 ± 58.00. The remaining variables did not give a significant contribution in building an efficient non linear model and were excluded from the final algorithm.

**Figure 4:**
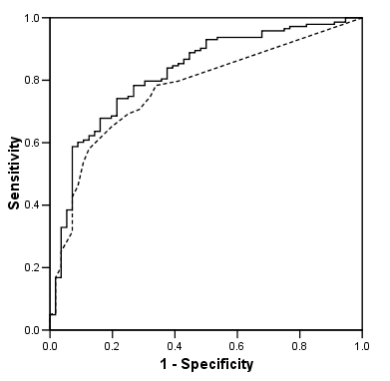
**Prediction of the follow-up haemoglobin**. ROC curves plotting sensitivity against 1 minus specificity for a cut-off of 11.0 g/dL in the prediction of the haemoglobin one month later obtained from the *nephrologists *(*dotted line*) and from the best performing *ANN *(*continuous line*) (using as input variables the haemoglobin and epoetin dose from the currently and the 2 previous months). The areas under the curves, the 95% confidence intervals and the significance *P *for the Nephrologists and the ANN were respectively: 0.772 (0.702–0.881), *P *< 0.001 and 0.822 (0.758–0.887), *P *< 0.001(the difference between the two curves was not significant).

The accuracy, the agreement and the *r*/NMSE ratio in predicting the haemoglobin of one month later (follow-up haemoglobin) by the nephrologists in charge of the patients and the best performing ANN (using as input variables the haemoglobin and epoetin dose from the current and the 2 previous months and being structured as a Generalized Feedforward Network with 6 hidden neurons) is depicted in Table 3. The prediction ability of both the nephrologists and the ANN is demonstrated using ROC curves in Figure 4 (haemoglobin cut-off 11.0 g/dL).

**Table 3:** Prediction ability of the nephrologists compared to ANNs

	**Nephrologists**	**ANN**
	
**Mean absolute error**	0.24	-0.02
**SD**	0.8972	0.8184
**CRMSE**	**0.9279**	**0.8186**
**LA**	-1.5577	-1.6519
	2.0311	1.6218
**95%CI bias**	0.1310	-0.1115
	0.3424	0.0814
**95% CI lower**	-1.7408	-1.8189
	-1.3746	-1.4849
**95% CI upper**	1.8480	1.4548
	2.2142	1.7889
**NMSE**	0.8020	0.6239
***r *Pearson**	0.5337	0.6135
***r*/NMSE**	**0.6654**	**0.9831**

Accuracy expressed by the Combined Root Mean Square Error (CRMSE) and agreement expressed by the "limits of agreement" (LA), the "95% confidence interval for the bias" (95% CI bias), the "95% confidence interval for the lower (95% CI lower) and upper (95% CI upper) limits of agreement", the Normalized Mean Squared Error (NMSE), the Pearson linear correlation *r *and the *r*/NMSE ratio in the prediction of the haemoglobin one month later.

The sensitivity, the specificity, the positive and negative predictive value in predicting a follow-up haemoglobin < 11.0 g/dL of the best performing ANN compared with the nephrologists were 0.48 (27/56) versus 0.25 (14/56) *P *< 0.05, 0.92 (132/143) versus 0.83 (119/143) *P *< 0.05, 0.71 (27/38) vs. 0.39 (14/36) *P *< 0.01 and 0.82 (132/161) vs. 0.73 (119/162) n.s. respectively.

## Discussion

This study was performed in order to characterize once more the linear and non-linear relationship between several clinical and biological variables and the response to epoetin beta and to test the performance of non-linear mathematical models, based on artificial neural networks, in the prediction of the erythropoietin dose required for an individual patient. Our results show that ANNs which could be implemented in wards are clearly superior to linear regressions or the nephrologist's opinion in predicting the erythropoietin dose for an individual patient.

The first observation of this study is that linear regressions are less performant than ANNs as predictive tools. Indeed, as illustrated in Figure 3, in contrary to non-linear mathematical models based on ANNs, the best performing linear regression did not demonstrate any significant ability to predict erythropoietin responsiveness. This performance gap confirms a non-linear relationship between factors influencing both erythropoietin responsiveness and need in individual patients. As highlighted in previous studies [41,42], on the basis of the results of the linear regressions, the administration route of erythropoietin significantly influenced the erythropoietin dose: patients treated with intravenous erythropoietin required a higher erythropoietin dose (16.0% more) and had lower haemoglobin (0.53 g/dL less).

Analysing the AIMS*EOC *data pool, the main variables influencing the mean erythropoietin dose needed to obtain a haemoglobin of 11.5 g/dL were weight, drug administration route (subcutaneous vs. intravenous), age and ferritin (see Figure 2 Panel A). Among these variables the relevance of the correlation with the weight has to be considered cautiously since the epoetin dose in the AIMS*EOC *database was indexed to body weight and in a previous study the absence of justification for body weight adjusted dosage was demonstrated [43]. Confirming previous epidemiological and experimental data, the analysis of the *EOC *subgroup allowed the identification of further variables showing a relevant non linear relationship: particular attention should be given to pH, Kt/V, PTH and CRP (see Figure 2 Panel B) [9,10,13-15].

The good performance of the ANN built using as input variables weight, epoetin administration route, age, presence or absence of cardiomyopathy and creatinine is shown in Figure 3 (ROC curves). For a specificity of 50%, the sensitivity of ANNs compared with linear regressions in predicting the erythropoietin dose to reach the haemoglobin target was 78 vs. 44% (*P *< 0.001). Considering that the cited variables are included in the blood tests usually performed before starting an epoetin substitution, additional information about erythropoietin responsiveness could be easy obtained without supplementary costs.

Curiously enough not one of the cited variables contributed significantly in the building of the model structured to predict the monthly adaptations in epoetin dose in individual patients. This means that, as suggested by pharmacodynamic models for other drugs [44] and as confirmed by the results of previous studies [24,26], the monthly fluctuations in haemoglobin as a function of the erythropoietin dose over a 3-month period are indirectly expressing all the other tested parameters related, in an analogous non linear mathematical model, to erythropoietin responsiveness. Of note, a large intra- and inter-individual variability in the requirements of erythropoietin (17.5 ± 19.2 and 35.3 ± 34.0 % respectively), to be referred at least in part to the inclusion in the study even of patients with intercurrent illnesses susceptible to influence the haemoglobin value, making the prediction of the ideal dose particularly difficult, was found in our database.

Compared with the nephrologists in charge of the patients, following the European best practice guidelines, the best performing ANN built to predict the monthly adaptations in epoetin dose on the basis of the haemoglobin and of the epoetin prescribed in the previous two months would allow the detection of 48 vs. 25% of the patients treated with an insufficient dose with a specificity of 92 vs. 83% (positive and negative predictive values 71 vs. 39 and 82 vs. 73% respectively). Only in 2 cases (0.8% of the tested group) the follow-up haemoglobin of the patients selected to be treated with a higher epoetin dose would have been > 12.0 g/dL (12.1 and 12.8 g/dL respectively) without adaptation in the dose. This finding, compared with the performance of the nephrologists in the same group (the epoetin dose would have been increased in 4 patients with a follow-up haemoglobin > 12.0 g/dL without adaptation in the dose), offers sufficient guarantees for the application of the selected ANNs in the clinical setting. Furthermore, compared with previous studies (Table 4), the present one was conducted on a larger multicentric group of patients (432 from 29 dialysis units) with a strong inter-individual variability. This fact should guarantee the applicability of the models beyond the studied population. However, considering that increasing the prediction power of the nephrologists is not the only condition needed to promote efficiency in erythropoietin prescription, the real impact of our computer assisted tool has to be evaluated in a prospective randomized trial.

**Table 4:** Comparative summary of the published studies

**Authors**	**Number of patients/centres included**	**Epoetin isoform**	**Variables explored (inputs)**	**Predictions (outputs)**
*Gabutti et al *(present study)	432/29	Beta	Sex; age; weight, presence or absence of a diabetes mellitus and/or a cardiomyopathy with EF<50%; haemoglobin; creatinine; BUN; pH; ionized calcium; albumin; CRP; ferritin; PTH; epoetin and iron dose; epoetin administration route sc vs. iv; Kt/V	Epoetin dose and follow-up haemoglobin
*Bellazzi *[24]	10/1	n.s.	Sex; age; haemoglobin; calcium; PTH; epoetin dose and others non specified	Follow-up haemoglobin
*Martin Guerriero et al *[20]	110/1	Alpha and beta	Age; weight; haemoglobin; ferritin; epoetin dose; isoform and number of administrations weekly; iron dose	Epoetin dose
*Gaweda et al *[25]	209/1	n.s.	Haematocrit; albumin; ferritin; iron saturation; PTH; epoetin and iron dose; Kt/V	Follow-up haematocrit
*Jacobs et al *[26]	166/n.s.	n.s.	Haematocrit; albumin; ferritin; iron saturation; PTH; epoetin and iron dose; Kt/V	Follow-up haematocrit

n.s.: not stated

Comparative table summarizing the characteristics of the studies already published predicting the epoetin dose and/or the follow-up haemoglobin/haematocrit with ANNs.

Coming back to the choice of using ANNs as a non linear adaptive learning machine for individualizing epoetin dosage regimens, their usefulness by clinical ward oriented nephrologists has been demonstrated once again. However even if compared to other computer assisted mathematical models for non linear adaptive modeling ANNs are easy to use and to access and tolerate both missing data and errors in individual variables well, their user friendliness contrasts with the still persisting difficulties in correctly evaluating the reliability of the obtained functions [34-36].

The next step will be to include in the electronic documentation of the dialysis patients in use in our centres individualized models automatically warning the nephrologists about the need and modality of adaptations in the epoetin dose.

## Conclusion

Compared with linear models, computer assisted tools based on artificial neural networks predict the mean erythropoietin dose required for an individual patient significantly better. Surprisingly using non linear correlations the most important variable influencing the epoetin requirement expressed in units/kg/week is the weight. Even if a particular attention should be reserved to both the pre-dialysis pH and the epoetin administration route, a prediction tool built with other variables known to influence the epoetin responsiveness will not increase in a quantitatively significant way the prediction power of the model.

As expected the model built to predict the dose adjustments is mainly influenced by the historical haemoglobin levels and even using only the data of the previous 2 months would allow compared with nephrologists, with a specificity of 92%, to detect a further 23% of the patients treated with an insufficient dose of erythropoietin.

Thus, implementing computer assisted tools that help predict the ideal erythropoietin dose, allowing timely and appropriate prescription adjustments, is an important challenge that will have relevant consequences on the patients' quality of life and should be further encouraged.

## Competing interests

The author(s) declare that they have no competing interests.

## Authors' contributions

LG conceived of the study, participated in its design and coordination, performed the statistical and data analysis and drafted the manuscript. NL and JB performed the data collection and participated in the data analysis and in drafting the manuscript. GM, CM and MB participated in the study design and data analysis and in drafting the manuscript. All authors read and approved the final manuscript.

## Pre-publication history

The pre-publication history for this paper can be accessed here:


